# High Performance Ternary Solid Polymer Electrolytes Based on High Dielectric Poly(vinylidene fluoride) Copolymers for Solid State Lithium-Ion Batteries

**DOI:** 10.1021/acsami.3c03361

**Published:** 2023-06-28

**Authors:** João C. Barbosa, Daniela M. Correia, Arkaitz Fidalgo-Marijuan, Renato Gonçalves, Stanislav Ferdov, Verónica de Zea Bermudez, Senentxu Lanceros-Mendez, Carlos M. Costa

**Affiliations:** † Physics Centre of Minho and Porto Universities (CF-UM-UP) and Laboratory of Physics for Materials and Emergent Technologies, LapMET, University of Minho 4710-057 Braga, Portugal; ‡ CQ-VR, University of Trás-os-Montes e Alto Douro, 5000-801 Vila Real, Portugal; § Centre of Chemistry, University of Minho, 4710-057 Braga, Portugal; ∥ BCMaterials, Basque Center for Materials, Applications and Nanostructures, UPV/EHU Science Park, 48940 Leioa, Spain; ⊥ Department of Organic and Inorganic Chemistry, University of the Basque Country UPV/EHU, 48940 Leioa, Spain; # Department of Chemistry, University of Trás-os-Montes e Alto Douro, 5000-801 Vila Real, Portugal; ¶ Ikerbasque, Basque Foundation for Science, 48009 Bilbao, Spain; ● Institute of Science and Innovation for Bio-Sustainability (IB-S), University of Minho, 4710-053 Braga, Portugal

**Keywords:** ternary composites, PVDF copolymers, lithium salt and ionic liquids, solid polymer electrolytes, lithium-ion batteries

## Abstract

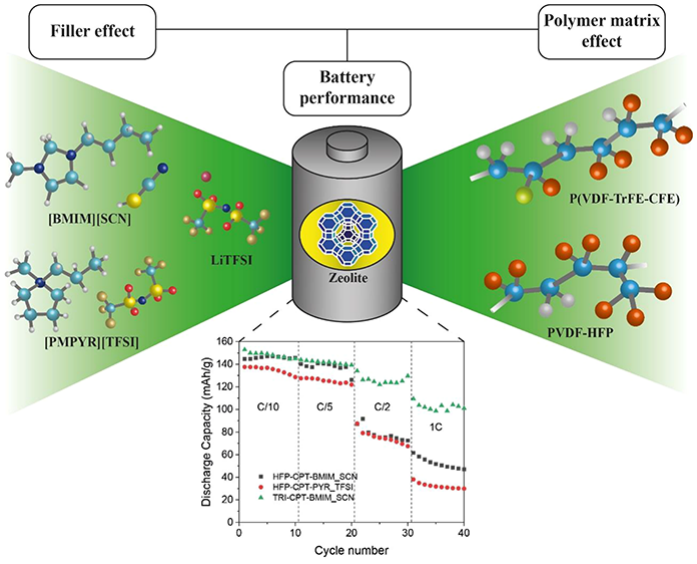

Renewable energy sources require efficient energy storage systems. Lithium-ion batteries stand out among those systems, but safety and cycling stability problems still need to be improved. This can be achieved by the implementation of solid polymer electrolytes (SPE) instead of the typically used separator/electrolyte system. Thus, ternary SPEs have been developed based on poly(vinylidene fluoride-*co*-hexafluoropropylene) (PVDF-HFP) and poly(vinylidene fluoride-trifluoroethylene-chlorofluoroethylene), P(VDF-TrFE-CFE) as host polymers, clinoptilolite (CPT) zeolite added to stabilize the battery cycling performance, and ionic liquids (ILs) (1-butyl-3-methylimidazolium thiocyanate ([BMIM][SCN])), 1-methyl-1-propylpyrrolidinium bis(trifluoromethylsulfonyl)imide ([PMPyr][TFSI]) or lithium bis(trifluoromethanesulfonyl)imide (LiTFSI), incorporated to increase the ionic conductivity. The samples were processed by doctor blade with solvent evaporation at 160 °C. The nature of the polymer matrix and fillers affect the morphology and mechanical properties of the samples and play an important role in electrochemical parameters such as ionic conductivity value, electrochemical window stability, and lithium-transference number. The best ionic conductivity (4.2 × 10^–5^ S cm^–1^) and lithium transference number (0.59) were obtained for the PVDF-HFP-CPT-[PMPyr][TFSI] sample. Charge–discharge battery tests at C/10 showed excellent battery performance with values of 150 mAh g^–1^ after 50 cycles, regardless of the polymer matrix and IL used. In the rate performance tests, the best SPE was the one based on the P(VDF-TrFE-CFE) host polymer, with a discharge value at C-rate of 98.7 mAh g^–1^, as it promoted ionic dissociation. This study proves for the first time the suitability of P(VDF-TrFE-CFE) as SPE in lithium-ion batteries, showing the relevance of the proper selection of the polymer matrix, IL type, and lithium salt in the formulation of the ternary SPE, in order to optimize solid-state battery performance. In particular, the enhancement of the ionic conductivity provided by the IL and the effect of the high dielectric constant polymer P(VDF-TrFE-CFE) in improving battery cyclability in a wide range of discharge rates must be highlighted.

## 1 Introduction

The global energy supply situation stated the urgent need for a transition to sustainable and reliable energy sources. However, the intermittency of renewable energy sources means that to be fully effective, they must be integrated with energy storage systems. Lithium-ion batteries (LIBs) are the dominant systems for this purpose nowadays and one of the main drivers of the electronic transition in the scope of the 4th industrial revolution. Their massive use is related to their high power and energy density, no memory effects, and durability, which are significant advantages when compared to previous technologies.^1^ These features allow their use for a variety of applications, from small and portable electronic devices to larger ones as electric vehicles and stationary energy storage systems. The main challenge of current LIB technology is its reliance on liquid electrolytes, which can degrade battery components and performance over time due to interface interactions. Further, liquid electrolytes are typically toxic, are easily flammable, and require strong encapsulation to avoid leakages, which can bring environmental and human risks.^2,3^

To overcome these issues, solid polymer electrolytes (SPEs) emerged as a potential solution, as they can simultaneously act as battery separator and electrolyte, eliminating the need for liquid components in the battery.^4^ Since the first works with poly(ethylene oxide) (PEO) and lithium salts,^5^ the SPE field has grown exponentially always focusing on overcoming the current limitations, which include their low ionic conductivity at room temperature and the limited interfacial compatibility with the electrodes.^6,7^ Usually, a SPE is composed of a polymer matrix, which can be poly(ethylene glycol) (PEG),^8^ poly(acrylonitrile) (PAN),^9^ PEO,^10^ poly(vinylidene fluoride) (PVDF) and copolymers,^11^ among others, and one or more fillers.^12^ The latter can act directly by improving the ionic conductivity of the system (active fillers) or indirectly, by enhancing other SPE properties, such as thermal or mechanical stability, further contributing to better SPE performance (passive fillers).^6^ Balancing the type and proportion of the different fillers is a critical challenge to achieve optimized SPE performance.^6^

The most common passive fillers are ceramics,^13^ carbonaceous^14^ materials, or microporous materials, such as metal–organic frameworks (MOFs)^15^ and zeolites.^16,17^

Regarding zeolites, they are attracting increasing interest due to their stabilization effect on a polymer matrix, particularly at the mechanical and thermal levels, leading to improved battery cycling stability.^6^ Clinoptilolite (CPT) is one of the most studied zeolites in this framework,^16^ due to its ion exchange capability, low density, high surface area, and large pore volume.^18,19^ Furthermore, CPT is a natural material, being cheaper to process than similar synthetic metal–organic framework (MOF) structures.

Regarding active fillers, lithium salts are the most widely used materials because they impart high ionic conductivity.^20^ Lithium bis(trifluoromethylsulfonyl)imide (LiTFSI) is the most extensively studied, showing effective results,^21,22^ due to its high ionic conductivity and thermal stability.^23^ A more recent approach is focused on the use of ionic liquids (ILs), as they also possess high ionic conductivity and ability to reduce the polymer crystallinity, further facilitating ion diffusion.^24^ When combined with fluorinated polymers, they induce the polymer crystallization in the polar β-phase, improving Li^+^ dissociation and reducing the SPE resistance.^25−27^ Their ability to bond to other materials, such as zeolites, is also an interesting feature to improve battery performance. In this regard, 1-butyl-3-methylimidazolium thiocyanate ([BMIM][SCN]) has been explored as one of the most compatible IL for this purpose.^17,28^ Another interesting IL is 1-methyl-1-propylpyrrolidinium bis(trifluoromethylsulfonyl)imide ([PMPyr][TFSI]) due to its high ionic conductivity and remarkable plasticizing effect.^29^

The polymer matrix is a host for the fillers and an insulator barrier. Recent studies showed that a high dielectric constant can have a positive effect on the ion conduction mechanism.^30^ Until now, the dielectric constant was improved using ceramic particles, such as BaTiO_3_, which poses limitations due to the increase in the system’s complexity.^31^ However, the development of a novel fluorinated terpolymer poly(vinylidene fluoride-trifluoroethylene-chlorofluoroethylene), P(VDF-TrFE-CFE), characterized by a high dielectric constant (ε′ = 40) and high chemical, thermal and mechanical stability, may provide a new generation of improved polymer matrix for SPE development.^32^

In previous works, the preparation method for ternary composites has been addressed in which zeolite type and ion exchange have been optimized.^17,28^ This work reports on the development and characterization of advanced SPEs by evaluating the effect of the fluorinated polymer matrix, poly(vinylidene fluoride-*co*-hexafluoropropylene) (PVDF-HFP) and the P(VDF-TrFE-CFE), which is introduced for the first time for this application, in combination with ion-exchange CPT as passive filler for stabilizing battery cycling performance, two different ILs ([BMIM][SCN] and [PMPyr][TFSI]) and a lithium salt, LiTFSI, as active fillers to optimize SPE overall performance. In all the SPEs developed, the polymer phase, morphology, electrical, thermal, and mechanical properties were evaluated. Further, the electrochemical performance for these SPE in solid-state lithium-ion batteries was investigated.

## 2 Experimental Section

### 2.1 Materials

**Figure 1:**
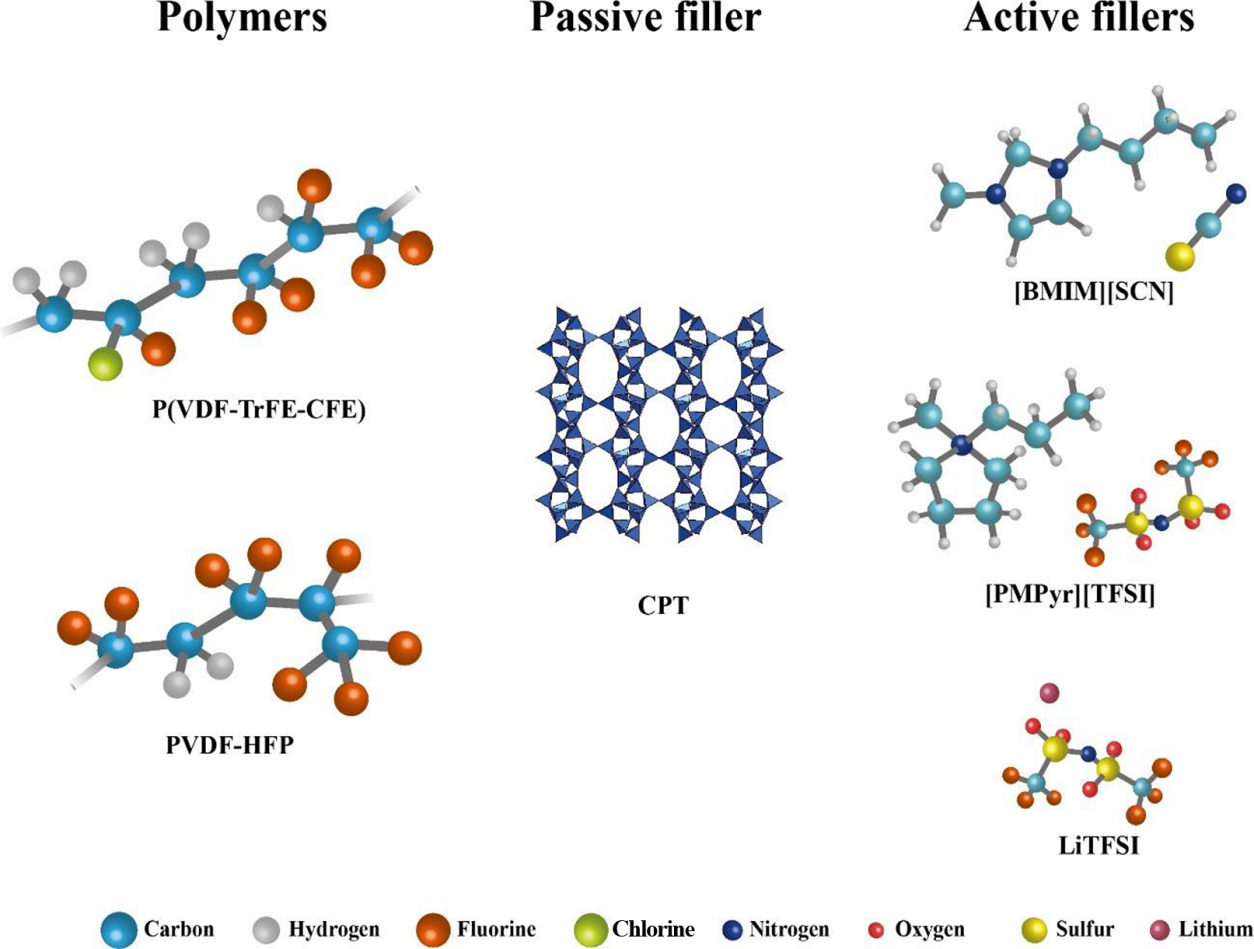
Chemical structures of the materials used in the SPE preparation.

PVDF-HFP (Kynarflex PVDF-HFP 2801-00107) and P(VDF-TrFE-CFE (Piezotech RT-FS) were obtained from Arkema-Piezotech. [BMIM][SCN] and [PMPyr][TFSI] were purchased from Iolitec. CPT and LiTFSI were acquired from Newstone International LLC, Japan, and Solvionic, respectively. A schematic representation of the materials used in the SPE preparation is presented in Figure 1. Lithium hydroxide (LiOH), *N*-methyl-2-pyrrolidone (NMP, 99%), and *N*,*N*-dimethylformamide (DMF, 99%) were purchased from Merck. Regarding the cathode electrode, LiFePO_4_ (LFP), Super P conductive additive, and PVDF (Kynar PVDF HSV900), they were supplied by Lithium Phostech, Timcal Graphite & Carbon, and Arkema, respectively.

### 2.2 Sample Preparation

First, the CPT zeolite was subjected to an ion exchange process as described in ref (28). 1 M LiOH solution was mixed with the CPT powder (50 mL of solution per 0.5 g of zeolite). This solution was then put in an oven (PSelecta) for 72 h at 90 °C. More LiOH solution was progressively added to compensate for the evaporation. Finally, the solution was filtered, and the powder was washed with distilled water. For removal of the residual water, the powder was dried overnight in an oven at 60 °C.

The SPE samples were prepared according to the preparation method reported in ref (6). First, the IL/LiTFSI was added to DMF (filler:polymer = 40:60 weight ratio) and mixed under magnetic stirring for about 30 min. Then, the polymer was added (polymer:solvent = 15:85 weight ratio) and mixed under magnetic stirring for about 2 h until complete polymer dissolution. Later, the ion exchanged CPT was added to the solution (filler:polymer = 16:84 weight ratio), which was put on an ultrasonic bath for 3 h to warrant a good dispersion of the particles. A glass substrate was used to cast the resulting solution using a doctor blade to reach a uniform thickness around 50 μm after solvent evaporation. The samples were put in an oven (PSelecta) at 160 °C for 15 min, as this is the optimal temperature to achieve better sample performance. For the sake of clarity, the ternary composites were named X-CPT-Y, where X represents the chemical formula of the polymer (PVDF-HFP or TER for P(VDF-TrFE-CFE)) and Y is the chemical formula of the active filler (LiTFSI or [BMIM][SCN] or [PMPyr][TFSI]).

### 2.3 Sample Characterization

A scanning electron microscopy (SEM) setup from Carl Zeiss AG (EVO 40 series) was used to evaluate the SPE surface and cross-section morphology at 10 kV. Previously, the SPE samples were deposited with a conductive gold layer by magnetron sputtering (Polaron model SC502). The cross-section was obtained by mechanically breaking the samples after a liquid nitrogen bath.

A Panalytical X’pert Cu Kα diffractometer was used to obtain the X-ray diffraction (XRD) patterns of the SPE samples in the range 2θ = 5–70°, exposure time of 10 s/step, and a step size of 0.015°. The degree of crystallinity (100 – % amorphous) of the samples was calculated (eq 1) through the DIFFRAC.EVA (Bruker, AXS) software, defining the amorphous and crystalline regions,^33^ the amorphous area being (global area – reduced area):
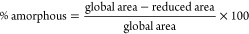
1

A Jasco FT/IR-6100 equipment was used for obtaining the Fourier transform infrared (FTIR) spectrum (attenuated total reflection (ATR) mode) from 4000 to 600 cm^–1^ at 4 cm^–1^ resolution and 64 scans. The polar phase (*F*(β)) content within the polymer was obtained through the eq 2:^34^
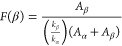
2where *A*_α_ and *A*_β_ are the absorbances at 766 and 840 cm^–1^, corresponding to the α and the β phases, respectively. *K*_α_ and *K*_β_ are the absorption coefficients for these specific bands, i.e, 6.1 × 10^4^ and 7.7 × 10^4^ cm^2^ mol^–1^, respectively.^34^

Differential scanning calorimetry (DSC) was carried out from 25 to 200 °C at 10 °C min^–1^ in a nitrogen atmosphere using PerkinElmer DSC 6000 equipment.

Thermogravimetric analysis (TGA) was achieved through a NETZSCH STA 449F3 thermobalance between 20 and 800 °C at 5 °C min^–1^ in a nitrogen atmosphere. The crucible contained about 10 mg of each sample.

The mechanical stress–strain curves were carried out with the TST350 tensile testing stage from Linkam Scientific Instruments. The measurements were obtained at a strain rate of 1 mm mim^–1^ at room temperature in SPE samples with approximate dimensions of 30 μm × 10 μm × 50 μm.

The ionic conductivity (σ_i_) values were obtained from electrochemical impedance spectroscopy through symmetry cells gold|SPE|gold electrodes with Autolab PGSTAT-12 (Eco Chemie) equipment. The SPE samples were previously heated at 60 °C in a Buchi TO51 tube oven. The Nyquist plots were obtained with amplitude of 10 mV at frequencies between 0.1 mHz and 10^6^ Hz from 25 to 80 °C. The ionic conductivity value of the SPE samples was calculated using eq 3:
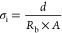
3where *d* is the SPE thickness, *R*_b_ is the bulk resistance, and *A* is the electrode area.

In these ternary SPEs, the ionic conductivity value is dependent on the temperature (*T*) and follows the Arrhenius equation in the measured range:
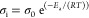
4where σ_0_ is a pre-exponential factor, *R* is the gas constant (8.314 J mol^–1^ K^–1^), and *E*_a_ is the apparent activation energy.

The electrochemical stability of the samples was obtained at room temperature by cyclic voltammetry at 0.1 mV s^–1^ (Autolab PGSTAT-12 (Eco Chemie)). The lithium metal|SPE|gold microelectrode (25 μm diameter) was used for the measurements, performed in a dry argon-filled glovebox.

The Bruce and Evans method was used to determine the Li-ion transference number (*t*_Li_^+^) by potentiostatic polarization using symmetrical lithium cells and applying a DC voltage of 10 mV.^35^ The *t*_Li_^+^ value was considered by eq 5:
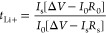
5where *I*_s_ is the steady current, Δ*V* is the applied potential, *I*_0_ is the initial current, *R*_0_ is the resistance of the Li electrode/electrolyte before polarization, and *R*_s_ is the resistance after polarization.

The SPE stability against lithium metal was also tested using symmetric Li||SPE||Li cells assembled under an argon atmosphere through evaluation by electrochemical impedance spectroscopy in an Autolab PGSTAT-12 (Eco Chemie) for 7 days.

### 2.4 Battery Testing

LFP cathodes were prepared with a weight ratio of 80/10/10 of active material/polymer binder/conductive material, with an active mass loading of about 2 mg. The electrode slurry was deposited in an aluminum current collector. More details on the preparation were reported in ref (36). Before the cathode materials and the SPE samples were transferred to the glovebox, they were dried overnight at 60 °C under vacuum (Buchi TO51 tube oven). The cathodic half-cells were then assembled under argon atmosphere (H_2_O, O_2_ <1 ppm) in a glovebox through this configuration: lithium metal|SPE|LFP cathode.

Galvanostatic cycles were obtained at C/10 rate (C = 170 mA g^–1^) for 50 cycles at room temperature (Landt CT2001A instrument). The battery performance of the SPEs was also evaluated at several rates (C/10, C/5, C/2, and 1C) for 10 cycles. Before and after cycling, an evaluation of the assembled batteries’ electrical properties was performed by impedance spectroscopy in the frequency range from 10 mHz to 500 kHz and a signal amplitude of 10 mV using an Autolab PGSTAT12.

## 3 Results and Discussion

### 3.1 Morphological Analysis

**Figure 2:**
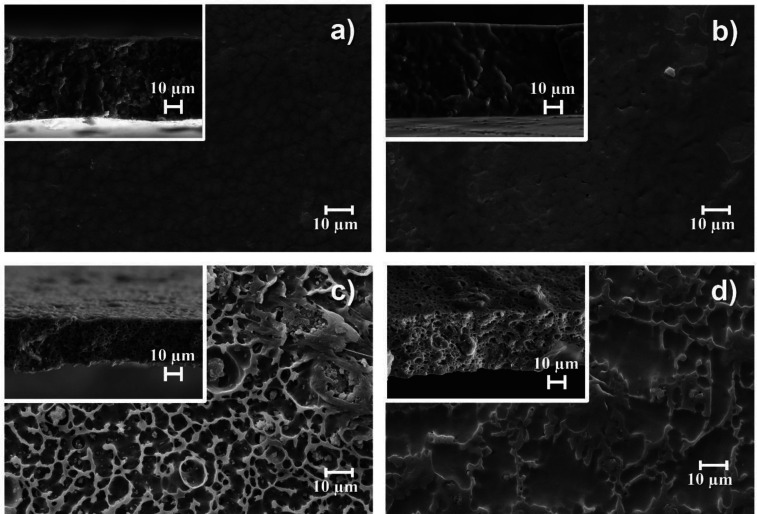
SEM surface and cross section images: (a) PVDF-HFP-CPT-[BMIM][SCN], (b) PVDF-HFP-CPT-[PMPyr][TFSI], (c) PVDF-HFP-CPT-LiTFSI, and (d) TER-CPT-[BMIM][SCN] samples.

The morphology of the SPE samples was evaluated by using SEM, as shown in Figure 2. The images of the surface of the samples show a good homogeneity and distribution of the ionic liquid, zeolite, and lithium salt indicating the compatibility between all components.

These findings lead us to conclude that the presence of the fillers leads to the inhibition of the spherulite growth, resulting in a large number of small spherulites throughout the samples.^37^ This is particularly visible in the case of the PVDF-HFP/IL samples (Figure 2a,b), as the ILs act as a nucleation agent for crystallization in the PVDF-HFP matrix.^25^ On the other hand, upon addition of the LiTFSI salt (Figure 2c), a homogeneous porous structure was formed due to a phase separation process during polymer crystallization, ascribed to the interaction of the Li^+^ salt with the solvent.^38^ A similar behavior is also visible for the P(VDF-TrFE-CFE) matrix, in contrast with the PVDF-HFP one for the same filler (CPT and [BMIM][SCN]), which presents a large microporous network along the cross-section (Figure 2d) due to a phase separation process ascribed to the different nature of the polymer matrix and its interaction with the solvent.^39^

**Figure 3:**
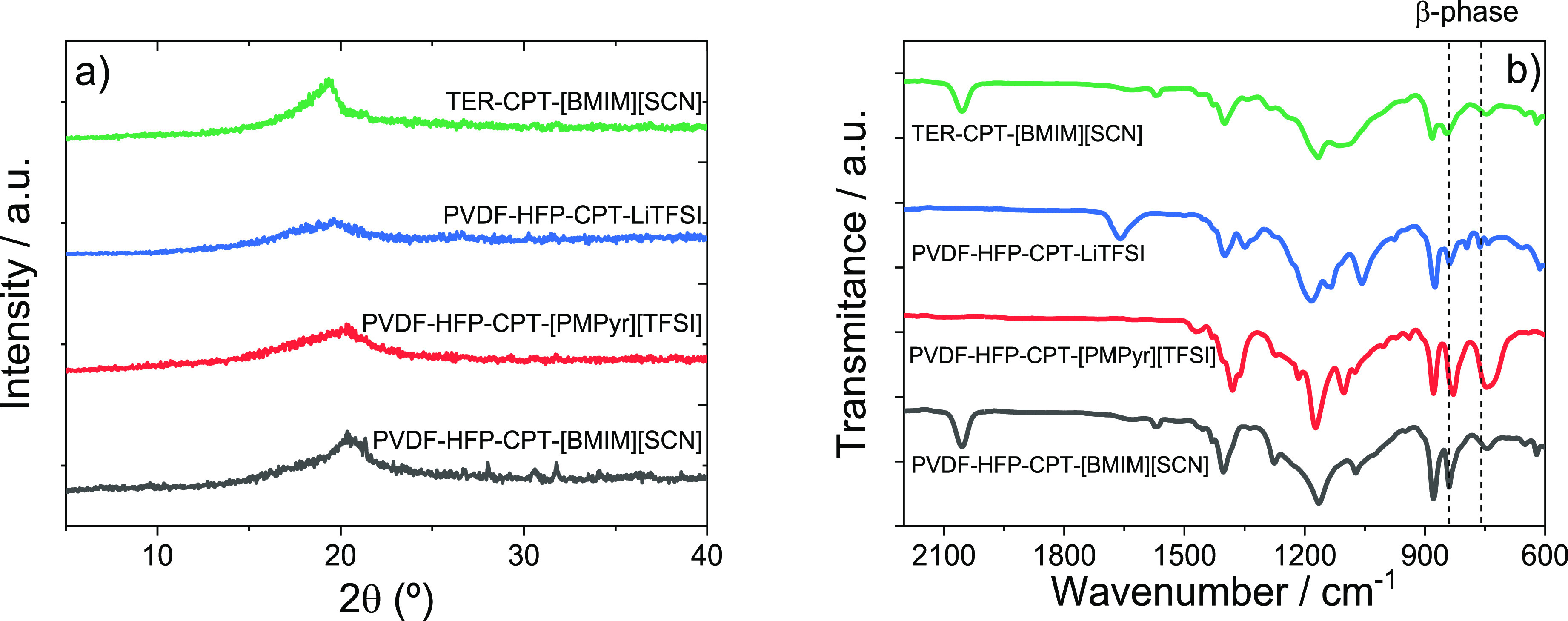
(a) XRD patterns and (b) ATR/FTIR spectra of the prepared samples.

Figure 3a shows the XRD patterns for the SPE samples, demonstrating a similar trend in all cases. In fact, the typical peaks of PVDF-HFP and P(VDF-TrFE-CFE) polymers at 2θ = 17.9, 18.6, 20.1, and 26.9° are not observed, due to the high filler loading in the samples.^34^ The crystallinity degree of the samples was determined from these XRD patterns, and the results are listed in Table 1. The crystallinity of the samples is independent of the IL and polymer matrix used, due to the high IL content that hinders polymer crystallization.^40^ In the PVDF-HFP-CPT-LITFSI sample, the crystallinity was further reduced due to the complexation of the salts by the polymer matrix.^41^

**Table 1:** Calculated Values for the Degree of Crystallinity and β-Phase Content of the Samples

sample	degree of crystallinity (% ± 2%)	β-phase (% ± 2%)
PVDF-HFP-CPT-[BMIM][SCN]	26	87
PVDF-HFP-CPT-[PMPyr][TFSI]	26	46
PVDF-HFP-CPT-LiTFSI	22	75
TER-CPT-[BMIM][SCN]	26	81

The influence of the fillers on the polymer chain conformation was analyzed by using ATR/FTIR spectroscopy (Figure 3b). The stretching vibrations of the CF_2_ and CH_2_ groups of the polymer are visible at 678, 763, 795, and 976 cm^–1^, regardless of the sample.^34^ The 840 and 760 cm^–1^ bands, which identify the polar β phase conformation of PVDF, show high intensity in the spectra, indicating the significant content of this phase in the samples. This behavior is confirmed by the β-phase content determination based on eq 1 (Table 1). The high β-phase content is attributed to the role of the ILs as nucleation agents, which lead to strong ion–dipole interactions which promote the crystallization of the polymers in the all-*trans* planar zigzag conformation.^40^ The low β-phase content value calculated for the PVDF-HFP-CPT-[PMPyr][TFSI] sample may be attributed to the overlapping of the characteristic α phase bands with those of the [PMPyr][TFSI] IL, which affects the phase calculation.

### 3.2 Thermal and Mechanical Properties

**Figure 4:**
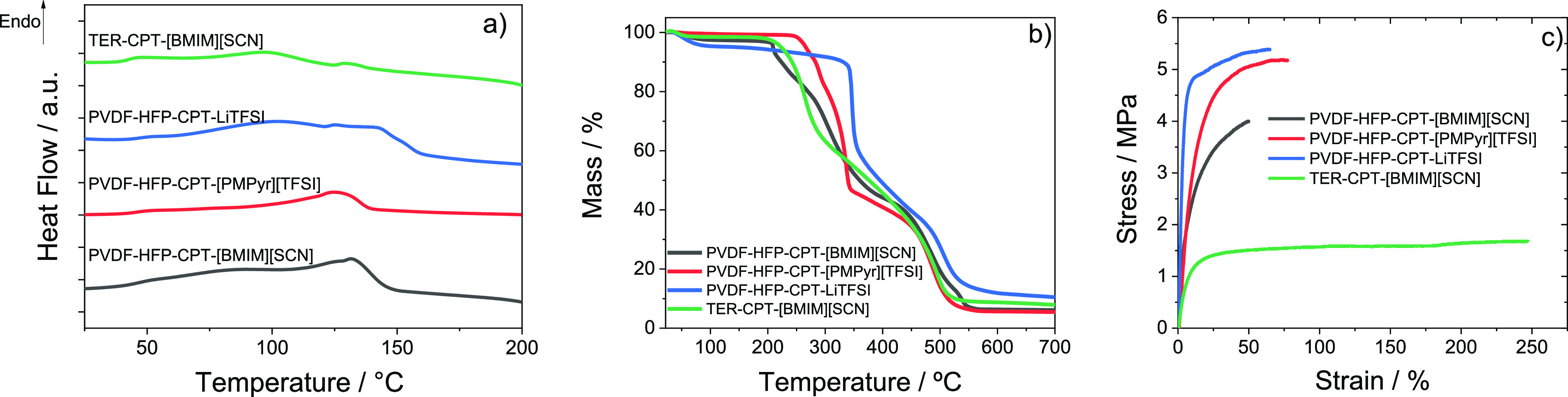
(a) DSC, (b) TGA, and (c) stress–strain curves of the prepared samples.

DSC analysis was used to evaluate the samples’ thermal behavior (Figure 4a). The polymer melting peak expected at about 145 °C is present in all samples,^17^ with a slight shift to lower temperatures due to the breakdown of the crystalline polar phase of the polymer, resulting from its electrostatic interactions with the IL.^42^ The exception is the PVDF-HFP-CPT-LiTFSI sample, in which the endothermic peak is shifted to higher temperatures due to the overlapping of the polymer melting peak with a LiTFSI solid–solid transition at 152 °C.^43^

Regarding the samples’ thermal degradation, TGA data (Figure 4b) show that all samples present distinct degradation steps corresponding to the different components. The PVDF-HFP and P(VDF-TrFE-CFE) degradation steps ascribed to the scission of carbon–hydrogen (C–H) bonds is overlapped with the degradation step of the CPT at about 475 °C.^44^ The degradation steps of the ILs are around 265 and 320 °C for [BMIM][SCN] and [PMPyr][TFSI], respectively. These values are slightly higher than those found in the literature due to the CPT interaction with the ILs previously reported,^17^ which delays their thermal degradation. The TGA curve of the PVDF-HFP-CPT-LiTFSI sample presents an extra mass loss below 100 °C associated with water evaporation from the salt structure, and the degradation step of the LiTFSI at around 350 °C, which is in line with the reported values.^45^

The mechanical curves of the SPE samples are presented in Figure 4c, showing the mechanical characteristic behavior of a thermoplastic polymer.^46^ In this curve, the elastic and plastic regions separated by the yield region are affected by the presence of the fillers. Comparing the prepared composite samples with their own pristine polymers PVDF-HFP and P(VDF-TrFE-CFE), it was verified that there was a decrease of the yield stress from 22 and 3 MPa for the pristine polymers to 4–5.5 and 1.5 MPa for the prepared composite samples, respectively. The mechanical tests show the typical mechanical reinforcement effect of the CPT due to the restriction of the polymer chain motion. As proven by the Young modulus values presented in Table 2,^47^ they were calculated using the tangent method, at a maximum deformation of 3% in the elastic region, and they are lower than those obtained for the pristine PVDF-HFP polymer.^17^ The Young modulus of the PVDF-HFP-CPT-LiTFSI sample is the highest among the prepared samples and is ascribed to the strongest interaction of the salt with the high dielectric polymer, leading to a more rigid response.^48^ Regarding the polymer matrix, the use of the P(VDF-TrFE-CFE) proves to significantly increase the mechanical stability of the samples, which are able to stretch more than 250% of their initial length without breaking, as previously observed.^32^

**Table 2:** Young Modulus and Yield Strength Values of the SPE Samples Obtained from the Mechanical Curves

sample	Young modulus, MPa (±5%)	yield strength, MPa (±5%)
PVDF-HFP-CPT-[BMIM][SCN]	59	1.54
PVDF-HFP-CPT-[PMPyr][TFSI]	39	1.05
PVDF-HFP-CPT-LiTFSI	92	3.37
TER-CPT-[BMIM][SCN]	12	0.59

### 3.3 Electrochemical Properties

**Figure 5:**
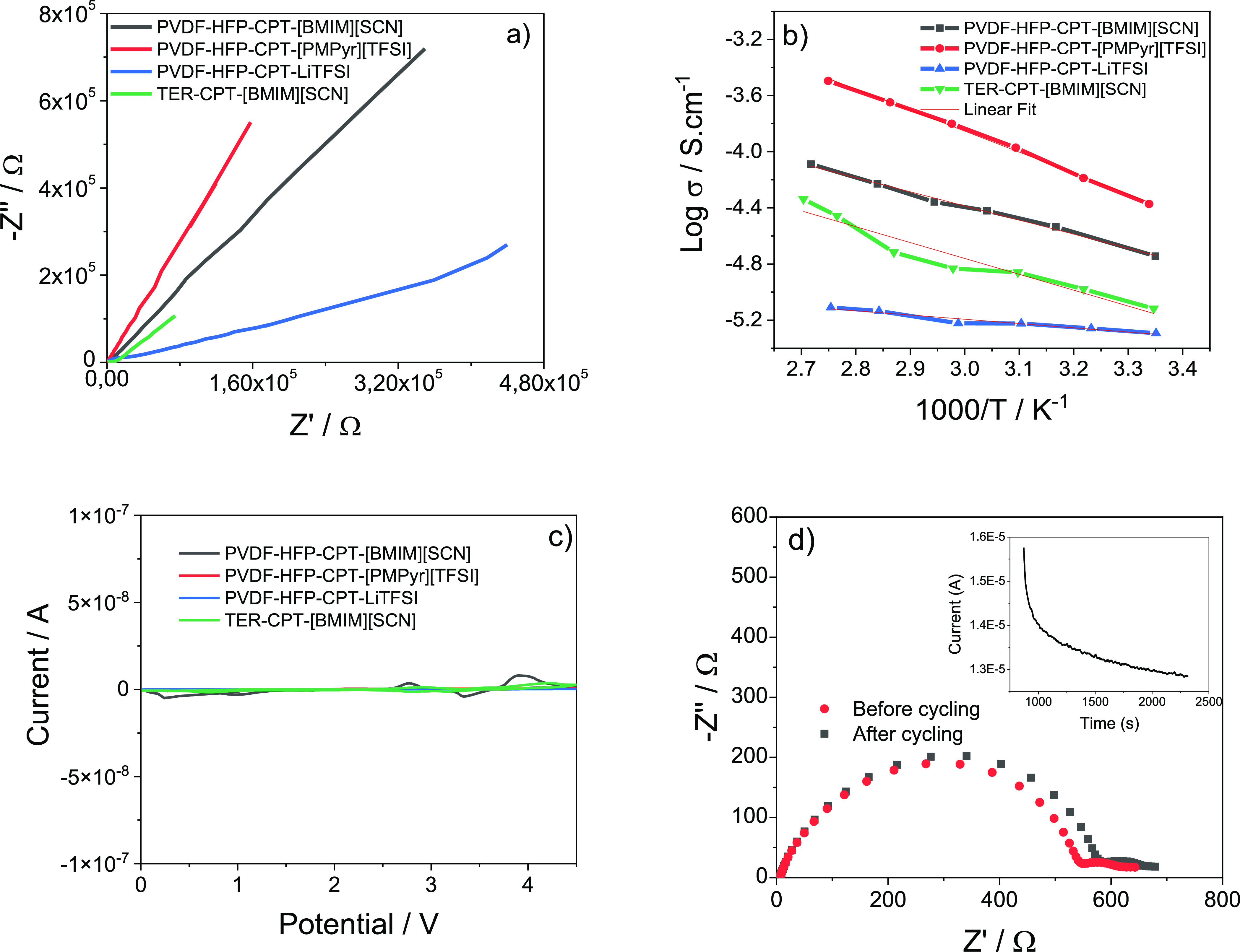
(a) Nyquist plots of the samples at room temperature. (b) Ionic conductivity value at different temperatures. (c) Cyclic voltammogram of the samples and (d) chronoamperometry of the sample with the highest Li^+^ transference number, PVDF-HFP-CPT-[PMPyr][TFSI].

Electrochemical characterization is an effective way to evaluate the suitability of a SPE to be applied in LIBs.^49^ The ionic conductivity was evaluated at different temperatures by electrochemical impedance spectroscopy. The Nyquist plots of the SPE samples measured at room temperature (Figure 5a) are characterized by the following regions: a semicircular zone at the high frequency end, related to the charge transfer process; an intermediate region associated with diffusion of counterions in the electrode; and at the lower frequencies, the straight line corresponding to the ions’ diffusion process.^50^

In this particular case, the semicircle is not visible, meaning that the main conduction mechanism in these samples is the diffusion of ions, represented by the straight line, which is attributed to the significant number of mobile charge carriers related to the IL and the Li^+^ salt.^51^ The ionic conductivity was calculated from the Nyquist plots at different temperatures, and the results are shown in the Arrhenius plots in Figure 5b. The increase of the ionic conductivity with temperature is observed due to the higher mobility of the ionic species and of the polymer chains.^39^ The levels of ionic conductivity are lower for the PVDF-HFP-CPT-LiTFSI sample due to the salt interaction with the polymer matrix resulting in the complexation of these components and to a limited number of free charges when compared to the ILs.^17^ Also, it is observed that the IL type and polymer matrix affect the ionic conductivity due to the distinct electrostatic interactions between the IL cations and anions and the polymer chains. The best ionic conductivity at room temperature was obtained for the PVDF-HFP-CPT-[PMPyr][TFSI] sample with a value of 4.2 × 10^–5^ S cm^–1^. The ionic conductivity values at room temperature and 60 °C for the SPE samples are presented in Table 3.

**Table 3:** Ionic Conductivity Values of Different Temperatures, Activation Energy Values, and Lithium Transference Number for All Samples

sample	ionic conductivity 25 °C, S·cm^–1^	ionic conductivity 60 °C, S·cm^–1^	activation energy, kJ mol^–1^	Li^+^ transference number
PVDF-HFP-CPT-[BMIM][SCN]	1.8 × 10^–5^	4.4 × 10^–5^	8.4	0.53
PVDF-HFP-CPT-[PMPyr][TFSI]	4.2 × 10^–5^	1.6 × 10^–4^	12.5	0.59
PVDF-HFP-CPT-LiTFSI	5.1 × 10^–6^	5.9 × 10^–6^	2.5	0.32
TER-CPT-[BMIM][SCN]	7.6 × 10^–6^	1.5 × 10^–5^	9.4	0.42

The thermal activation energy, calculated from the Arrhenius equation, are similar for samples (Table 3) and in line with those reported in literature for related systems.^28^ The exception is the PVDF-HFP-CPT-LiTFSI sample, for which the activation energy is lower due to the interaction of the charged species with the polymer matrix.

Further electrochemical analysis was carried out using cycling voltammetry to evaluate the electrochemical stability of the samples under different voltage conditions. There are no significant peaks observed for any sample in the voltage range of battery operation as shown in Figure 5c, indicating their stability for the application.

The lithium transference number was calculated following the Bruce–Vincent method,^52^ and the obtained values are presented in Table 3. There seems to be a correlation between the lithium transference number and ionic conductivity for the different samples, as they vary in the same way. The highest value of 0.59 is obtained for the PVDF-HFP-CPT-[PMPyr][TFSI], due to higher charge mobility of the IL compared to lithium salts, as shown in Figure 5d. High values near or above 0.5 are a good indicator of the Li^+^ diffusion capacity in the prepared samples, proving their suitability for applications in LIBs.

### 3.4 Battery Performance

**Figure 6:**
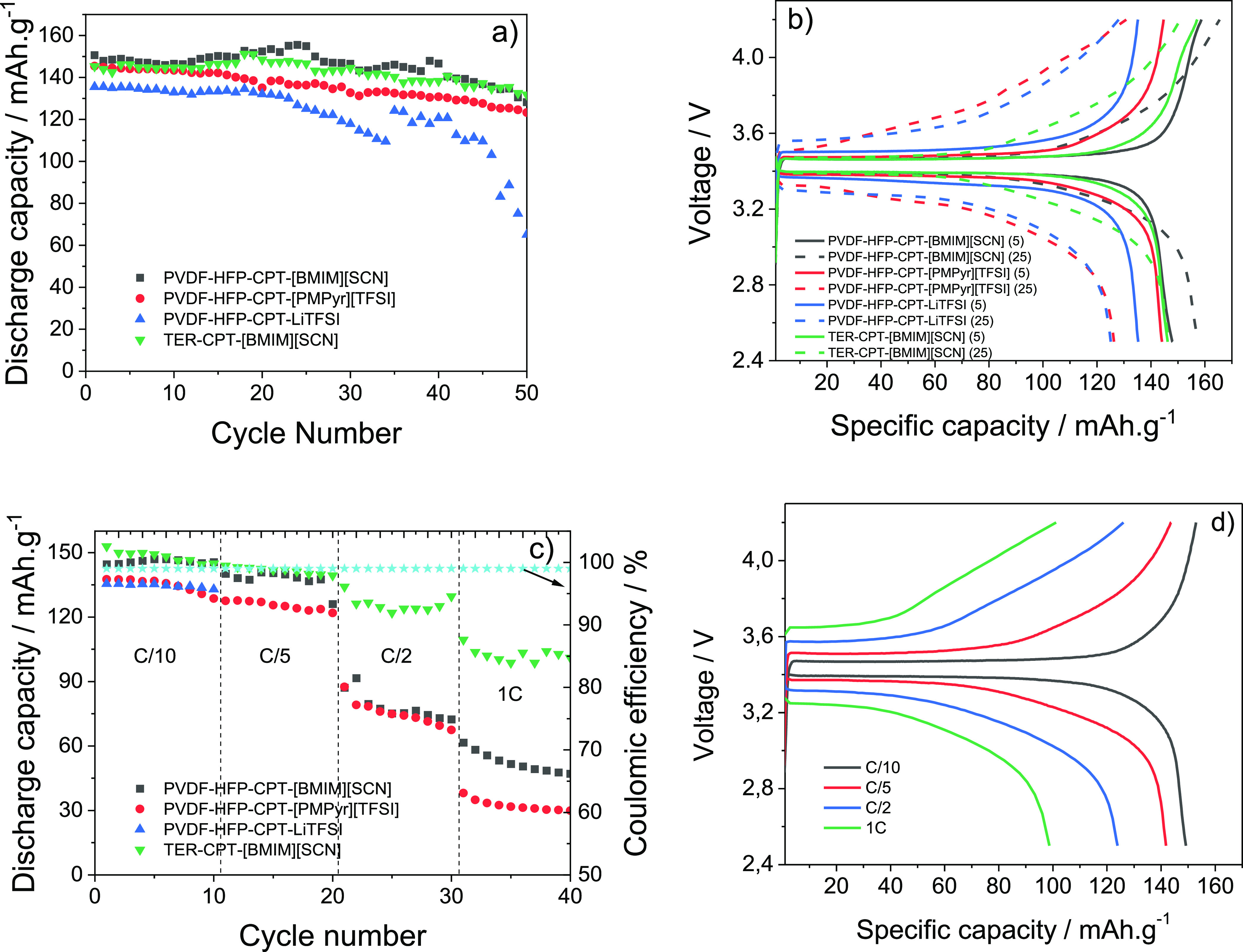
(a) Battery performance at room temperature for the different SPEs; (b) charge/discharge profile at the 5th and 25th cycles; (c) rate performance at C/10, C/5, C/2, and 1C rates; (d) charge/discharge profile of the TER-CPT-[BMIM][SCN] battery on the 5th cycle for each rate.

The prepared samples were assembled in cathodic half-cells to study their performance as SPEs for LIBs. The results obtained for the prepared half-cells are shown in Figure 6. The cycle life tests at C/10 (Figure 6a) show good stability for all samples during 50 cycles with values of about 150 mAh g^–1^ and a capacity retention between 85 and 90% except for the PVDF-HFP-CPT-LiTFSI one that significantly loses its capacity after 30 cycles. This behavior is correlated with the Coulombic efficiency, which drops significantly for this sample after the mentioned 30 cycles (Figure S1).

This is attributed to the strong complexation of the LiTFSI salt with the polymer matrix,.^17^ At this scan rate, the IL and polymer matrix interaction does not hinder battery performance. A more detailed analysis of the charge/discharge profiles of the assembled batteries between 2.5 and 4.2 V at the 5th and 25th cycle is shown in Figure 6b. The voltage plateau commonly observed in the LFP active material is attributed to the redox reaction of Fe^2+^/Fe^3+^, which corresponds to the extraction and insertion of Li^+^ ions within the structure. This voltage plateau is present regardless of the sample.^53^ The better performance at the 25th cycle, evidenced for the PVDF-HFP-CPT-[BMIM][SCN] sample, is attributed to the fact that the system is not fully activated at the 5th cycle, due to the formation of the SEI. For the other samples, the TER-CPT-[BMIM][SCN] seems to be the most stable one, this effect being ascribed to the use of a high dielectric constant polymer that contributes to increase the battery stability and ionic mobility.^30^

Considering the battery performance at the C/10-rate and to evaluate the effect of IL type and polymer matrix, Figure 6c shows the rate performance tests, where the TER-CPT-[BMIM][SCN] presents an outstanding performance, even at high discharge rates (nearly 100 mAh g^–1^ at 1C rate), which is much better when compared with those of the PVDF-HFP-CPT-[BMIM][SCN] and the PVDF-HFP-CPT-[PMPyr][TFSI] ones, with values of about 50 and 30 mAh g^–1^, respectively, for the same rate. This behavior is due to the high dielectric constant of this polymer, which promotes ionic dissociation, leading to lower ohmic polarization and improving battery performance. The PVDF-HFP-CPT-LiTFSI sample was not able to cycle at high discharge rates, so the results are not presented. The analysis of the charge/discharge profiles of the TER-CPT-[BMIM][SCN] at the 5th cycle of each rate (Figure 6d) clearly demonstrates its high stability, with values of 149.1, 141.8, 123.9, and 98.7 mAh g^–1^ for the C/10, C/5, C/2, and 1C rates, respectively, which represent 99%, 95%, 83%, and 66% of its initial discharge capacity, presenting a Coulombic efficiency of nearly 100% for all cycles. This makes this sample a particularly well-suited candidate for application in fast charging LIBs. The Coulombic efficiency shown in Figure 6c and indicating the reversibility of the process is high regardless of the cycle number and scan rate. The irreversible capacity observed as a function of the cycle number is explained by the cathodic decomposition, leading to the SEI formation.^54^

**Figure 7:**
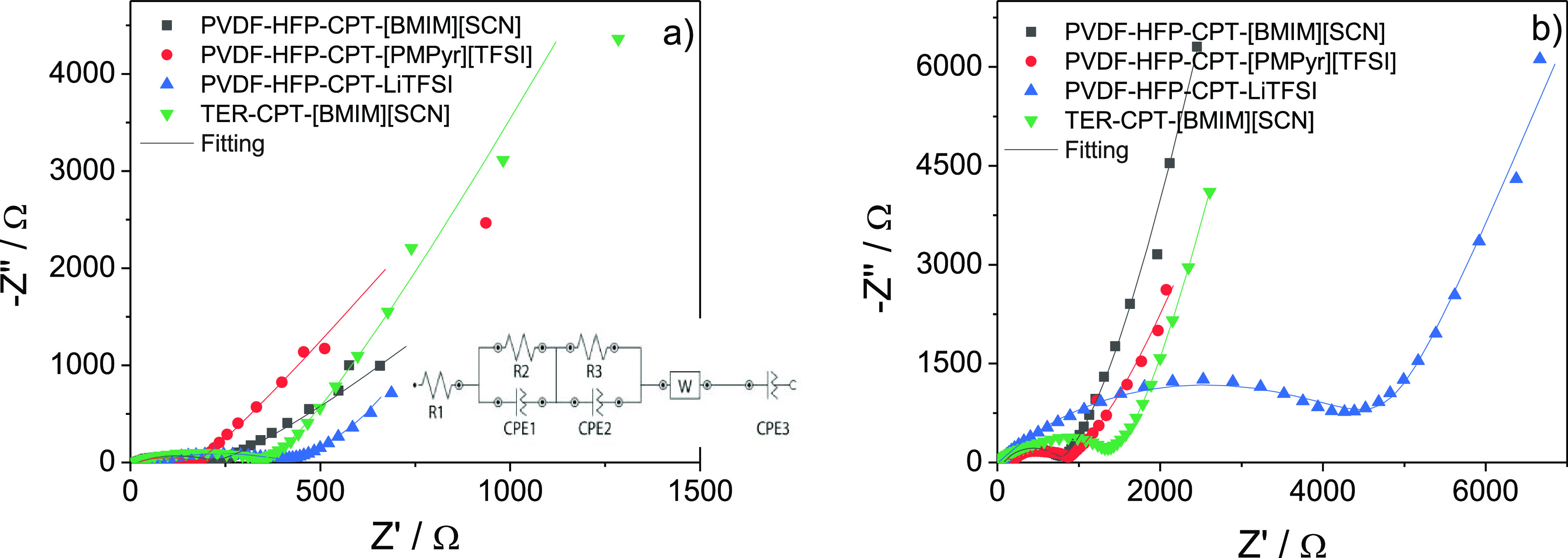
Nyquist plots of the assembled batteries at room temperature (a) before and (b) after cycling.

The reasons for the observed behavior can also be revealed by analyzing the impedance spectroscopy of the batteries (Figure 7). The increase in the resistance of the TER-CPT-[BMIM][SCN] sample after cycling is not significant when compared with the PVDF-HFP-CPT-LiTFSI one, which indicates that the SEI formation does not have a substantial impact on the battery performance in this case.^55^

Nyquist plots were obtained before and after battery cycling, and the results are presented in Figure 7. Both plots are characterized by a semicircle in the high frequency regions, which characterizes the battery’s overall resistance. This resistance is attributed to the ohmic resistance, resistance contributions from charge-transfer reactions, and contact film resistance. Further, a straight line in the low frequency region is associated with the Li^+^ diffusion process. Figure 7 also shows that the resistance increases after cycling is mainly due to the formation of the SEI layer.^55^

The samples’ behavior can be evaluated through the equivalent circuit displayed in the inset of Figure 7a, where the overall resistance (RT) is the combination of the contact film resistance (R2) and the resistance contributions from the charge-transfer reaction resistance (R3), in addition to the ohmic resistances (R1). The SPE sample with the lowest resistance before cycling is PVDF-HFP-CPT-[PMPyr][TFSI] and the sample with best performance is TER-CPT-[BMIM][SCN], for which the overall resistance before and after cycling is 342 and 1350 Ω, respectively. This means that in this sample, the increase in the resistance associated with the SEI growth is less significant, hinting a higher compatibility with the electrodes. This is further proven by the compatibility between this sample and the lithium metal evaluated for 7 days and presented in Figure S2. The cell resistance is increased from 316 to 1398 after 7 days, being an indicator of the high compatibility of the TER-CPT-[BMIM][SCN] based SPE with the lithium as the resistance value is below 1500 Ω.

This work proves the suitability of the three-component approach as a valid option for application in LIBs as SPEs. The obtained results are comparable or superior to those reported in the literature (Table 4), in particular that concerning TER-CPT-[BMIM][SCN], which possess the best high-rate cycling capacity at room temperature reported up to now. Comparing with other polymers such as PEO, the samples described in this work present a smaller lithium transference number but a comparable battery performance, which in the present case is achieved at room temperature. Similar to the obtained results, smaller lithium transference numbers are obtained for PVDF-HFP with embedded lithium salts.

**Table 4:** Comparison between the Obtained Results and Those Reported in the Literature

polymer	doping agents	conductivity (S·cm^–1^)	Li^+^ transference number	battery capacity of LFP batteries (mAh·g^–1^)/C-rate	ref.
PEO	SSZ-13, LiTFSI	5.34 × 10^–2^ (70 °C)	0.85	154 (C/10); 60 °C	(16)
PEO	ZYNa Zeolite, LiTFSI	1.66 × 10^–2^ (60 °C)	0.84	152 (C/5); 60 °C	(56)
PAES-co-PEG	[PYR][TFSI], LiTFSI	7.2 × 10^–4^ (25 °C)	0.38	139.7 (C/10); 25 °C	(57)
PEGMA, CTA	[PYR][TFSI], LiTFSI	5.24 × 10^–3^ (25 °C)	0.43	125 (C/20); 25 °C	(58)
PVDF	LiClO_4_, LLTO	5.8 × 10^–4^ (25 °C)	0.80	152 (C/5); 25 °C	(59)
PVDF-HFP	IL@UiO-67	4.3 × 10^–4^ (25 °C)	0.45	118 (1C); 25 °C	(60)
PVDF-HFP	LiTFSI, LLZTO	8.80 × 10^–5^ (25 °C)	0.27	158.7 (C/10); 25 °C	(26)
PVDF-HFP	CPT, [BMIM][SCN]	1.8 × 10^–5^ (25 °C)	0.53	147.9 (C/10); 25 °C	This work
PVDF-HFP	CPT, [PMPyr][TFSI]	1.6 × 10^–4^ (25 °C)	0.59	145.4 (C/10); 25 °C	This work
P(VDF-TrFE-CFE)	CPT, [BMIM][SCN]	7.6 × 10^–6^ (25 °C)	0.42	149.1 (C/10); 25 °C	This work

**Figure 8:**
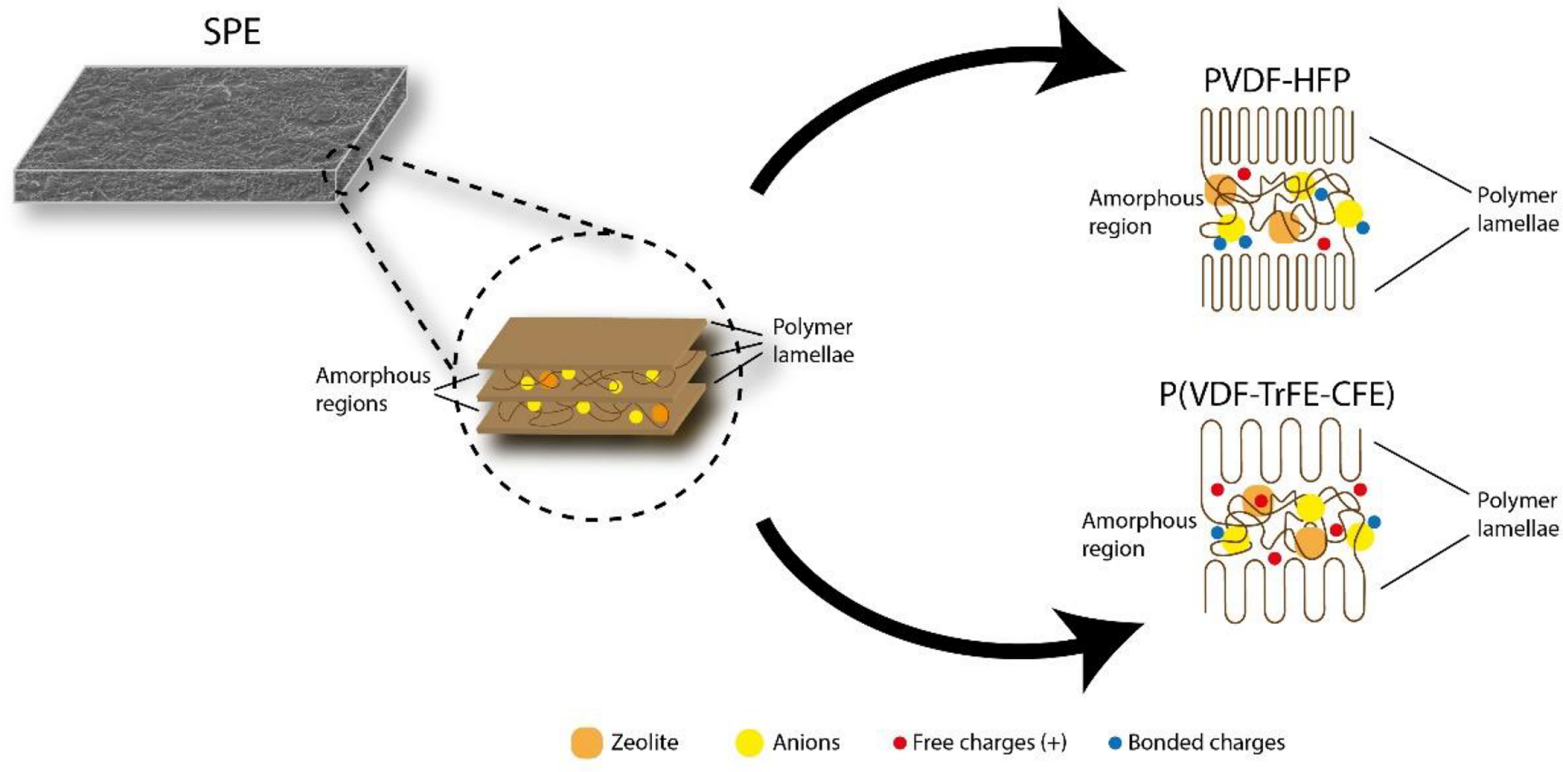
Schematic representation of the polymer/charge interaction in the lower (PVDF-HFP) and higher (P(VDF-TrFE-CFE)) dielectric constant polymers used in the present work.

In any case, we draw the attention to the fact that all the prepared samples are capable of delivering suitable capacities, which confirms the importance of selecting the right combination of materials, production methods, and conditions in order to optimize battery performance. It is of particular interest that even though the samples prepared in this work show lower ionic conductivity than other samples reported in the literature, the assembled batteries still show outstanding results, meaning that ionic conductivity alone is definitely not the most critical parameter in battery performance but instead a relevant factor in a more complex system. The important role of polymer selection in the final battery performance is also proven in this work, the high dielectric constant polymer leading to improved battery stability at high discharge rates. When compared to PVDF-HFP, the high dielectric constant P(VDF-TrFE-CFE) polymer presents an increased polymer interchain distance that weakens the intermolecular interactions, supports charge dissociation, and facilitates free charge motion through the SPE, as schematized in Figure 8.^61^

## 4 Conclusion

New solid polymer electrolytes (SPEs) of ternary composites based on poly(vinylidene fluoride-*co*-hexafluoropropylene) (PVDF-HFP) and poly(vinylidene fluoride-trifluoroethylene-chlorofluoroethylene), P(VDF-TrFE-CFE) as a polymer host, clinoptilolite (CPT) zeolite for stabilizing cycling performance and ionic liquids (IL) (1-butyl-3-methylimidazolium thiocyanate ([BMIM][SCN])), 1-methyl-1-propylpyrrolidinium bis(trifluoromethylsulfonyl)imide ([PMPyr][TFSI]), and lithium bis(trifluoromethanesulfonyl)imide (LiTFSI) for improving ionic conductivity, were produced by the doctor blade technique. The effect of the polymer matrix, different ILs and a lithium salt on the SPE morphology and thermal, mechanical, and electrical properties, was analyzed. The microstructure of the SPEs depends on the polymer matrix and fillers, ranging from compact for PVDF-HFP-CPT-[BMIM][SCN] to porous for PVDF-HFP-CPT-LiTFSI, being determined by the interaction between polymer chains, the fillers, and the solvent. The polymer matrix and the different fillers do not have a strong effect on the polymer phase, degree of crystallinity, or thermal degradation profile of the samples. Regarding the mechanical properties, the Young modulus is affected by the IL type, lithium salts, and polymer matrix due to the interaction of polymer chains and fillers. The highest ionic conductivity value (4.2 × 10^–5^ S·cm^–1^) and the highest lithium transference number (0.59) were obtained for the PVDF-HFP-CPT-[PMPyr][TFSI] sample.

The room temperature charge–discharge behavior at C/10 shows excellent battery performance with 150 mAh g^–1^, regardless of the IL type and polymer matrix. For rate performance tests, the highest battery performance was achieved for the poly(vinylidene fluoride-trifluoroethylene-chlorofluoroethylene), P(VDF-TrFE-CFE) SPE due to its high dielectric constant that promotes the dissociation of the ions and consequently the improvement of battery performance. The discharge values of this SPE sample are 149.1, 141.8, 123.9, and 98.7 mAh·g^–1^ for the C/10, C/5, C/2, and 1C rates, respectively, which represents 99%, 95%, 83%, and 66% of its initial capacity, with a Coulombic efficiency of almost 100% for all cycles.

It has been shown that the polymer matrix and filler type used for SPEs development affect the cycling behavior due to compatibility and interaction between IL and polymer matrix and that a high dielectric constant polymer matrix promotes ion dissociation and allows improvement of the performance of room temperature solid-state batteries.

## Supporting Information Available

Coulombic efficiency of the assembled cells andi mpedance of Li|electrolyte|Li symmetric battery (PDF)
