# Supplementary material for: High Performance Ternary Solid Polymer Electrolytes Based on High Dielectric Poly(vinylidene fluoride) Copolymers for Solid State Lithium-Ion Batteries
Source: ACS Appl Mater Interfaces. 2023 Jun 28;15(27):32301–12. doi: 10.1021/acsami.3c03361 (PMC10347132; doi:10.1021/acsami.3c03361)
Supplement: Supplementary file 1 — am3c03361_si_001.pdf [file am3c03361_si_001.pdf]

Supporting information

High Performance Ternary Solid Polymer Electrolytes Based on High Dielectric Poly(Vinylidene Fluoride) Copolymers for Solid State Lithium-Ion Batteries

João C. Barbosa[1,2], Daniela M. Correia[3], Arkaitz Fidalgo-Marijuan[4,5], Renato Gonçalves[3], Stanislav Ferdov[1], Verónica de Zea Bermudez[2,6], Senentxu Lanceros-Mendez[1,4,7*], Carlos M. Costa[1,8*]

[1]Physics Centre of Minho and Porto Universities (CF-UM-UP) and Laboratory of Physics for Materials and Emergent Technologies, LapMET, University of Minho 4710-057 Braga, Portugal

[2]CQ-VR, University of Trás-os-Montes e Alto Douro, 5000-801 Vila Real, Portugal

[3]Centre of Chemistry, University of Minho, 4710-057 Braga, Portugal

[4]BCMaterials, Basque Center for Materials, Applications and Nanostructures, UPV/EHU Science Park, 48940 Leioa, Spain.

[5]Department of Organic and Inorganic Chemistry, University of the Basque Country UPV/EHU, 48940 Leioa, Spain.

[6]Department of Chemistry, University of Trás-os-Montes e Alto Douro, 5000-801 Vila Real, Portugal

[7]Ikerbasque, Basque Foundation for Science, 48009 Bilbao, Spain

[8]Institute of Science and Innovation for Bio-Sustainability (IB-S), University of Minho, 4710-053 Braga, Portugal

*** Corresponding Authors**

Carlos M. Costa (cmscosta@fisica.uminho.pt)

Senentxu Lanceros-Méndez (senentxu.lanceros@bcmaterials.net)

S-1: Coulombic efficiency

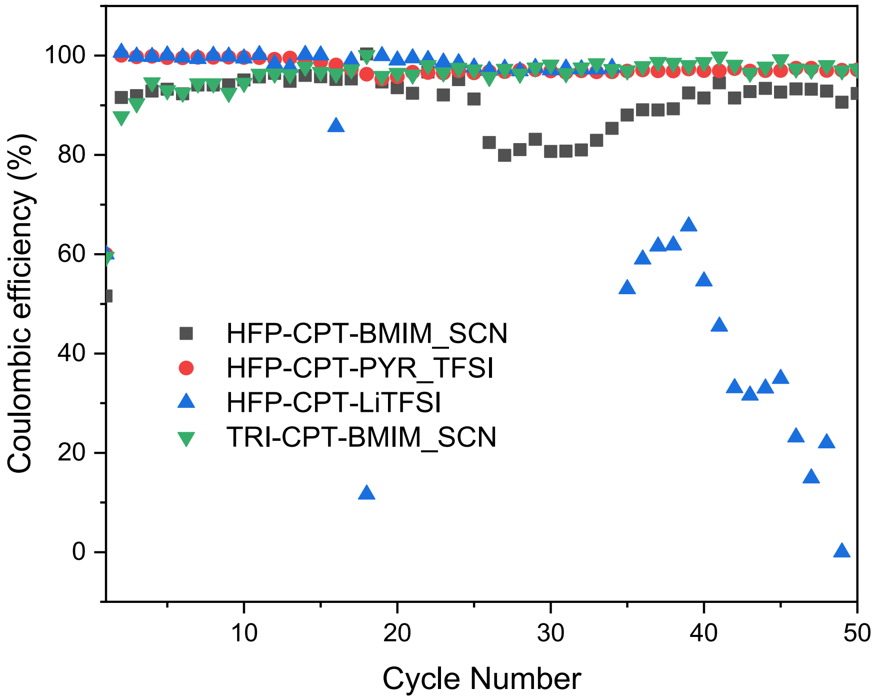

Figure S1. Coulombic efficiency of the assembled cells.

S-2: Li symmetric cell

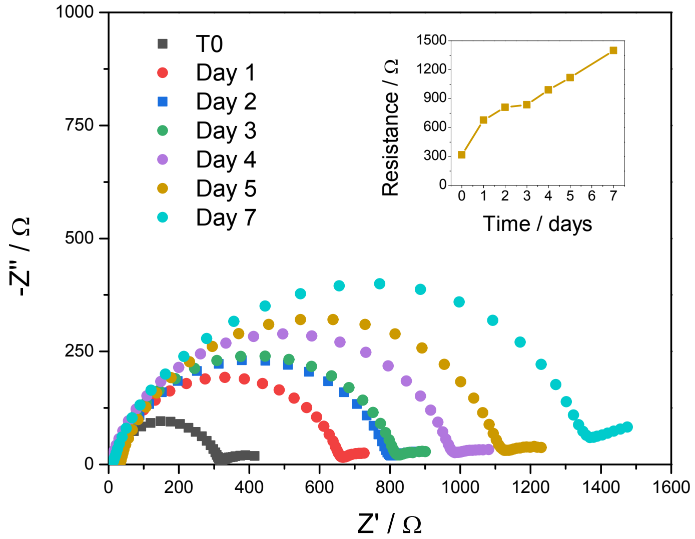

Figure S2. SPE stability for a Li symmetric cell during 7 days.
